# Virtual Water Flow Pattern in the Yellow River Basin, China: An Analysis Based on a Multiregional Input–Output Model

**DOI:** 10.3390/ijerph19127345

**Published:** 2022-06-15

**Authors:** Xiuli Liu, Rui Xiong, Pibin Guo, Lei Nie, Qinqin Shi, Wentao Li, Jing Cui

**Affiliations:** 1 Research Institute of Resource-Based Economics, Shanxi University of Finance & Economics, Taiyuan 030006, China; lxl820113@163.com (X.L.); xr1181968091@163.com (R.X.); nielei5515@foxmail.com (L.N.); shiqinqin_1314@126.com (Q.S.); liwentao0793@163.com (W.L.); cuijing000906@163.com (J.C.); 2 Department of Management, Taiyuan University, Taiyuan 030032, China

**Keywords:** virtual water flow, multiregional input–output model, pull index, water stress index, Yellow River Basin

## Abstract

Research on the Yellow River Basin’s virtual water is not only beneficial for rational water resource regulation and allocation, but it is also a crucial means of relieving the pressures of a shortage of water resources. The water stress index and pull coefficient have been introduced to calculate the implied virtual water from intraregional and interregional trade in the Yellow River Basin on the basis of a multi-regional input–output model; a systematic study of virtual water flow has been conducted. The analysis illustrated that: (1) Agriculture is the leading sector in terms of virtual water input and output among all provinces in the Yellow River Basin, which explains the high usage. Therefore, it is important to note that the agricultural sector needs to improve its water efficiency. In addition to agriculture, virtual water is mainly exported through supply companies in the upper reaches; the middle reaches mainly output services and the transportation industry, and the lower reaches mainly output to the manufacturing industry. Significant differences exist in the pull coefficients of the same sectors in different provinces (regions). The average pull coefficients of the manufacturing, mining, and construction industries are large, so it is necessary to formulate stricter water use policies. (2) The whole basin is in a state of virtual net water input, that is, throughout the region. The Henan, Shandong, Shanxi, Shaanxi, and Qinghai Provinces, which are relatively short of water, import virtual water to relieve local water pressures. However, in the Gansu Province and the Ningxia Autonomous Region, where water resources are not abundant, continuous virtual water output will exacerbate the local resource shortage. (3) The Yellow River Basin’s virtual water resources have obvious geographical distribution characteristics. The cross-provincial trade volume in the downstream area is high; the virtual water trade volume in the upstream area is low, as it is in the midstream and downstream areas; the trade relationship is insufficient. The Henan and Shandong Provinces are located in the dominant flow direction of Yellow River Basin’s virtual water, while Gansu and Inner Mongolia are at the major water sources. Trade exchanges between the midstream and downstream and the upstream should be strengthened. Therefore, the utilization of water resources should be planned nationwide to reduce water pressures, and policymakers should improve the performance of agricultural water use within the Yellow River Basin and change the main trade industries according to the resource advantages and water resources situation of each of them.

## 1. Introduction

Water is not only a necessary and irreplaceable resource for social and economic development [1,2,3], but it is also a vital element of the environment, essential for global sustainable development [4,5,6]. Water resources mainly refer to freshwater resources on land [5,7], with the total amount of freshwater available for human use accounting for only 0.3% of all freshwater. Since the 1990s, global water resources have been deteriorating [8,9,10]. About 1.5 billion people, accounting for 40% of the global population, in 80 countries and regions are suffering from a shortage of freshwater [11,12], and about 300 million from 26 countries are in an extreme water-shortage state [13,14,15]. Agricultural development will be especially hindered, and world food security will be compromised as a result of water shortages [16,17]. Globally, industrial water consumes approximately 20% of the total freshwater [18]; the shortage of water resources may lead to industrial shutdowns and limit production [19]. In addition, the destruction of ecosystems and biodiversity due to the water crisis will pose serious threats to human survival [20,21,22].

In terms of countries with water shortages, China is one of them [23,24]. Currently, China only possesses 6% of the global water resources [25], followed by Brazil, Russia, and Canada [26]. Nevertheless, China has a per capita water resource of only 2300 m^3^ [27], making it one of the most water-scarce countries in the world [28,29]. The unbalanced distribution of water resources is currently one of the biggest obstacles to promoting sustainable development in China [23,24]. There are significant differences in the distribution of water resources between North and South China [30,31,32]. For example, the Yellow River Basin covers an area of 795,000 km^2^ [33], accounting for only 44.2% of the Yangtze River Basin [27]. At present, the per capita water resources of 6 provinces (regions) in China are less than 500 m^3^, and water from the Yellow River Basin serves two-thirds of these provinces (regions) (Ningxia Autonomous Region, Henan Province, Shanxi Province, and Shandong Province) [34]; the shortage of water resources has become a major restricting factor for the Yellow River Basin’s high-quality development [35,36], posing quite a severe challenge to the construction of an ecological civilization and regional sustainable development [37,38]. The Yellow River Basin provides important water-resource support for China’s granary and national energy security, and it is tasked with supplying water to Hebei, Tianjin, the Jiaodong Peninsula, and other basins. The Yellow River Basin, which accounts for 2% of the river runoff in China, supports the water demands of 12% of the population and 17% of the arable land in China, and it plays a decisive role in the overall economic and social development. Therefore, quantifying the flow laws and operation trends of water resources used in economy and trade will have important theoretical value, and enhancing the Yellow River Basin’s intensive water usage is of practical significance [39,40,41], as is promoting its high-quality development in consideration of the water-resources carrying volume [42,43].

To better broaden the field of water research and find solutions for water scarcity in arid regions, Allen [44] proposed the concept of virtual water in 1993. The concept of virtual water illustrates that water resources, as a whole, required in various productions reflect the real quantity of resources in various economic production activities [44]. Virtual water has become one of the major methods for investigating regional water-resource issues [45]. Currently, the main method for studying virtual water is the input–output method, which can analyze trade and flows in a wide range of industries, and it is widely used by researchers globally [46,47,48]. Research on virtual water has multiple scales. First, the calculation model of trade and usage is established through the method of input and output, and then the volume of virtual water in any industrial sector is calculated [49,50]. Cegar [51] utilized the model discussed in this research to find both the indirect and the direct volumes of virtual water in the economy of Croatia; the water volume mainly relies on the processes of power generation and the utilization of the power processing output, along with the chemical and petroleum sectors. Gkatsikos and Mattas [52] analyzed water scarcity in Mediterranean countries and found that the agricultural sector dominated the regional virtual water flux. By analyzing the water usage of each link in commodity supply in China, Houyin et al. [53] found that the industrial sector was the core of indirect water usage. In regard to the continuous input and output table of Liaoning Province from 2012 to 2018, Zhang et al. [54] illustrated that the outflow sector of virtual water in Liaoning Province was mainly concentrated on primary and tertiary employment. Yang et al. [55] and Fu et al. [56] utilized this model to investigate the virtual water trade between the Tarim River Basin and Hubei Province in China, finding that the volume used of virtual water in the primary industry is much higher [32]. Zhang et al. [57] calculated both the direct and total water input coefficients in Inner Mongolia in 2007, 2012, and 2015. The research concluded that the direct and primary, secondary and tertiary total water input coefficients of the industries show a downward trend, indicating that the tertiary industry’s efficiency is constantly improving. Second, with the help of an input–output multi-regional model, the virtual water flux is combined with the economic relations among regions, so as to calculate the flow direction and usage of virtual water throughout all the regions. Islam et al. [58] conducted a multi-regional input and output analysis of virtual and direct water in 5 Australian capital cities and their surrounding areas, showing that the virtual part from outside the Australian cities boundary was nearly 20 times that from inside the urban boundary. Qasemipour et al. [59] assessed Iran’s virtual water flux via a multi-regional input–output framework (MRIO). The results showed that there was no shortage of water resources in the northern countries, and virtual water is imported through the trade in various products, while areas with serious water shortages are net exporters of virtual water. As pointed out by Dong et al. [60], Chen et al. [61], and Wang and Chen [62], virtual water in China presents a flow pattern from the inland to the coastal areas, as well as from the underdeveloped to the developed areas. Based on the analysis of water trade between provinces and interprovincial flows in Northeast China, Zhang et al. [63] suggested that Liaoning and Jilin Provinces have almost scarce water resources, and other regions have the highest cumulative risk scores of virtual water-trade spillovers compared with Liaoning Province. Third, with the increasing frequency of economic activities, virtual water flow also has had a certain impact on the utilization of water resources. Some scholars combine virtual water with water-resource utilization to study virtual water. Wang et al. [64] assessed the water security in five countries in Central Asia, finding that Tajikistan and Kyrgyzstan are relatively safe in terms of quantitative water-resource security, while Uzbekistan is at risk. Zhang et al. [65] analyzed and calculated water-resource usage efficiency in the Aral Sea region from 2000 to 2014. The results demonstrated that the Aral Sea’s water region dropped by 60.28% from the original 28,119 m^2^ over those 15 years. Through the accounting of virtual water in some areas of China, Zheng et al. [66] and Wang et al. [67] found that the utilization structure of water resources in the study area was unreasonable, and the utilization efficiency of water resources needed to be improved. Other scholars have also conducted investigations from the perspective of water resource pressures. For example, Rosales-Asensio E. et al. [68] noted the fact that the restrictions on water resources in the Canary Islands of Spain led to the over-exploitation of aquifers and wells, leading to the deterioration of water resources and the environment. De O et al. [9] found that, due to the expansion of irrigation areas and urban populations in the Rio Verde Grande Basin, Brazil, the availability of water resources was low, causing water-resource conflicts to be triggered. Gohar A. et al. [69] studied the groundwater resources in Barbados and concluded that, in order to protect the sustainability of aquifers, it was necessary to formulate policies to restrict pumping, while economic welfare would be reduced by a certain amount in a short time. The Chinese mainland, as the research area, was comprehensively evaluated for its regional water-resource pressures using virtual water flow by Sun et al. [70]. The results showed that the northeast and the Huang-Huai-Hai regions in China are the largest producers of food and the biggest exporters of virtual water, and the pressure of resource shortages is generally serious. Liu et al. [71] analyzed a water pressure index of 11 administrative regions from 2000 to 2013, including Hebei Province, China, where the demand and supply are in serious conflict. The results show that water stress is mainly manifested at three levels: high, medium, and low. The aforementioned studies generally argue that the shortage of water resources has become the main problem affecting regional sustainable development.

According to the analysis above, it can be seen that: (1) With regard to virtual water, the majority of the calculations focus on the use of a single regional or interprovincial industrial sector, while flows are less often studied from a regional or industrial-sector perspective; (2) Some studies focus on the fair distribution of water resources across regions, but they fail to fully combine the flow of virtual water with water resource management; (3) The research on key economic regions is not deep enough, especially those with serious shortages. Most studies focus on the virtual water in a single province in a basin; however, only a few studies have investigated the flow of virtual water within and between the Yellow River’s regions. The Belt and Road, the important node and the trade link between the Yellow River and China, is gradually strengthening under the current situation of double-cycle development in China. It is of great significance for water resource management strategy to analyze and study the virtual flow pattern implied by the Yellow River Basin’s internal and external trade.

To sum up, this paper takes the Yellow River regions as the object of this research, along with the interregional input and output data of a total of 31 provinces (municipalities and autonomous regions) domestically in 2015 and the water-usage data of 42 industrial sectors, constructs a multi-regional input–output accounting framework of the Yellow River Basin, and calculates the volume of virtual water trade within and between regions of the Yellow River Basin, respectively. Moreover, the pull coefficient and water stress index are introduced to further explore the coordination between the virtual water trade volume and local resource carrying capacity in the Yellow River region, as well as providing more-feasible policy suggestions for the management of the Yellow River region’s water resources.

In contrast to previous studies, the innovations of this paper are as follows: (1) Virtual water flow is discussed from the perspective of internal and external regions and sectors, which presents a theoretical basis for the formulation of systematic and rational regional trade policies and industrial water policies; (2) The introduction of the water stress index and pull coefficient to further investigate the dependence of the studied subject implied by regional trade on water resources locally and the tie between various sectors have practical significance for regions and sectors to formulate reasonable water-resource policies. This study provides a systematic and reasonable industrial and trade policy framework for optimizing the Yellow River region’s water resource allocations, promoting the protection of the aquatic ecological environment and alleviating its resource pressures.

The rest of the article is structured as follows: Section 2 introduces the research field, including the results and a virtual water analysis of an accounting framework regarding multi-regional input–output data and data sources pertaining to the Yellow River Basin. Section 3 presents the results and analysis. Section 4 puts forward a discussion, and suggestions are made in the conclusion.

## 2. Methodology and Materials

### 2.1. Study Region

**Figure 1:**
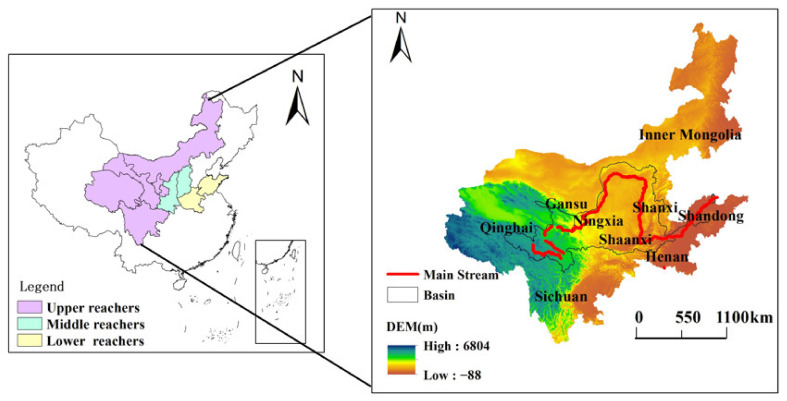
Locations of the upper, middle, and lower reaches of the Yellow River Basin (source: Authors’ own calculation/conception, using ArcGIS 10.7 software (Environmental Systems Research Institute, Inc., Taiyuan, China)).

The Yellow River region covers seven provinces and two autonomous regions, including the Shandong, Henan, Qinghai, Gansu, Sichuan, Shanxi, and Shaanxi Provinces, as well as the Ningxia and Inner Mongolia Autonomous Regions. It is one of the largest economic comprehensive zones in northern China [72,73]. According to the characteristics of the Yellow River Basin, the nine provinces are divided into three regions: upstream, midstream, and downstream [36,38,39] (Figure 1).

A total of 18.63% of the total domestic water resources came from the Yellow River Basin in 2019, and China’s Yellow River Basin GDP accounted for 25.08% nationally. The total quantity of water resources is basically balanced with the level of economic development (Table 1). Yet, economic development and water resources in the Yellow River Basin lack coordination. Upstream resources account for 82.32% of the basin’s total, while the GDP accounts for only 32.04%; the total quantity of downstream water-resource usage accounts for 6.72% of the Yellow River Basin, but it brings more than 50% of the GDP of the whole basin. In terms of total water usage, excepting Sichuan Province, the provinces (regions) located in the Yellow River Basin’s upper and middle reaches do not exceed 20 billion m^3^, and the downstream is much lower than the upstream. Therefore, encouraging the rational movement of water between the Yellow River Basin and the rest of the world is of paramount importance.

### 2.2. Multi-Regional Input–Output Virtual Water Accounting Framework of the Yellow River Basin

#### 2.2.1. Single-Region Input–Output Model

Since Leontief [74] proposed the input–output method, scholars globally have widely used this method due to its ability to analyze the direct and indirect usage of virtual water in various industrial sectors and regions. This chart illustrates the direct and indirect relationships between different regions and industries [75,76,77]. According to the input–output method, there is a balance between the output and usage of an economic system as follows:

(1) $$\left[\begin{array}{c} x_{1}\\ x_{2}\\ \vdots \\ x_{n} \end{array}\right]=\left[\begin{array}{cccc} z_{11} & z_{12} & \cdots & z_{1n}\\ z_{21} & z_{22} & \cdots & z_{2n}\\ \vdots & \vdots & \ddots & \vdots \\ z_{n1} & z_{n2} & \cdots & z_{nn} \end{array}\right]\left[\begin{array}{c} 1\\ 1\\ \vdots \\ 1 \end{array}\right]+\left[\begin{array}{c} f_{1}\\ f_{2}\\ \vdots \\ f_{n} \end{array}\right]$$

where $x_{i}^{r}$ is the total output of sector i in region r, $z_{ij}$ is the intermediate input provided by sector i to sector j, and $f_{i}$ is the final use of sector i.

The direct input coefficient $a_{ij}$ reflects the number of products and/or services directly consumed by each product per unit of complete output in the process and operation process of a product sector. Its calculation formula is:

(2) $$a_{ij}=z_{ij}/x_{j}$$

where $x_{j}$ is the total output of sector j.

Therefore, Equation (1) can be rewritten as:

(3) $$\left[\begin{array}{c} x_{1}\\ x_{2}\\ \vdots \\ x_{n} \end{array}\right]=\left[\begin{array}{cccc} a_{11} & a_{12} & \ldots & a_{1n}\\ a_{21} & a_{22} & \ldots & a_{2n}\\ \vdots & \vdots & \ddots & \vdots \\ a_{n1} & a_{n2} & \cdots & a_{nn} \end{array}\right]\left[\begin{array}{c} x_{1}\\ x_{2}\\ \vdots \\ x_{n} \end{array}\right]+\left[\begin{array}{c} f_{1}\\ f_{2}\\ \vdots \\ f_{n} \end{array}\right]$$

#### 2.2.2. Modeling of Yellow River Basin Inputs and Outputs on a Multi-Regional Scale

**Figure 2:**
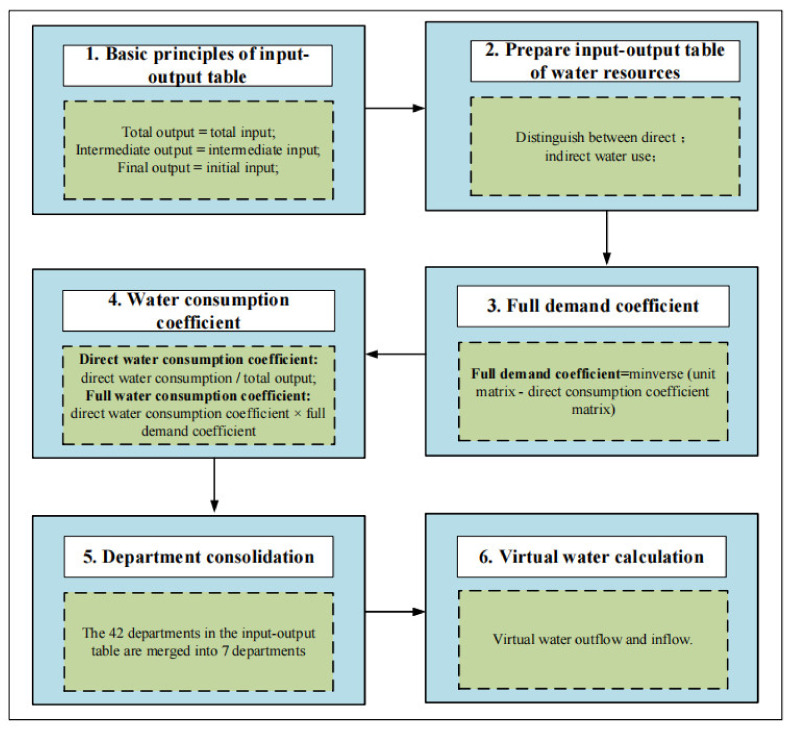
Model structure of virtual-flow-driving mechanism in the Yellow River Basin.

As is known to all, the multi-regional input–output method is able to connect the economies inside and outside a region with virtual water flow [49]. Therefore, in this paper, the calculation of provinces’ water usage was performed based on a multi-regional input–output method of analysis (regions) in the Yellow River region. According to the flow direction and geographical location characteristics of the Yellow River region, this paper defines the Yellow River region’s 9 provinces (regions) as local and the other 22 provinces (areas and cities) in China as foreign, leading to the construction of a multi-regional input–output table of the Yellow River Basin in 2015 (Table 2), which includes 10 regions and 42 industrial sectors in each region. Figure 2 is the input–output flow chart of this study.

Table 2 shows the multi-regional inputs and outputs of the Yellow River Basin; the balance of Regional r economic activities is:

(4) $$x_{i}^{r}=\overset{10}{\underset{s=1}{\sum }}\overset{42}{\underset{j=1}{\sum }}a_{ij}^{rs}x_{j}^{s}+\overset{10}{\underset{s=1}{\sum }}f_{i}^{rs}+e_{i}^{r}$$

where $x_{i}^{r}$ is the total output of sector i in region r, $a_{ij}^{rs}$ is the direct input coefficient, which indicates the direct input of sector i in region r to the production unit product of sector s in region j. $f_{i}^{rs}$ is the input of sector i in region r to the final demand of region s, and $e_{i}^{r}$ is the export volume of sector i in region r.

Equation (4) is expressed by the matrix as:

(5) $$X^{r}=A^{rs}+F^{rs}+E^{r}$$

Additionally, Equation (5) is appropriately reformed to obtain the multi-regional input–output model of the Yellow River Basin:

(6) $$X^{r}={\left(I-A^{rs}\right)}^{-1}\left(F^{rs}+E^{r}\right)=L\left(F^{rs}+E^{r}\right)$$

Among them,

(7) $$L={\left(I-A^{rs}\right)}^{-1}=\left[\begin{array}{cccc} q^{11} & q^{12} & \ldots & q^{1,10}\\ q^{21} & q^{22} & \ldots & q^{2,10}\\ \vdots & \vdots & \ddots & \vdots \\ q^{10,1} & q^{10,2} & \ldots & q^{10,10} \end{array}\right]$$

where $X^{r}$ is the total output matrix, I represents the identity matrix, and $A^{rs}$ means the direct input coefficient matrix. $F^{rs}$ and $E^{r}$ mean the end-use matrix and export matrix, respectively. $L={\left(I-A^{rs}\right)}^{-1}$ is the Leontief inverse matrix, and the element $l_{ij}^{rs}$ in the matrix donates the total output per unit product provided by sector i in region r to sector j in region s, where the regional direct water uses coefficient matrix Y, expressed as:

(8) $$Y=\left[y^{1},y^{2},\cdots ,y^{10}\right]$$

The complete water usage coefficient matrix Q is:

(9) $$Q=YL=Y{\left(I-A^{rs}\right)}^{-1}=\left[\begin{array}{cccc} q^{11} & q^{12} & \ldots & q^{1,10}\\ q^{21} & q^{22} & \ldots & q^{2,10}\\ \vdots & \vdots & \ddots & \vdots \\ q^{10,1} & q^{10,2} & \ldots & q^{10,10} \end{array}\right]$$

where the element $q^{rs}$ is the total usage of r area consumed by each sector in s area.

#### 2.2.3. Estimation of Virtual Water Trade Flow

According to the multi-regional input–output model of the Yellow River Basin, the virtual water trade flow among the nine provinces (regions) can be calculated as follows:

(10) $${\mathrm{VWT}}^{\mathrm{rs}}=\overset{9}{\underset{i=1}{\Sigma }}q^{is}f^{is}$$

The virtual water trade flow between the 9 provinces (regions) in the Yellow River region and other places is:

(11) $${\mathrm{VWI}}^{S}=\overset{10}{\underset{i=1}{\Sigma }}q^{10,i}f^{is}$$

(12) $${\mathrm{VWO}}^{r}=\overset{10}{\underset{i=1}{\Sigma }}q^{ri}f^{i,10}$$

where $VWT^{rs}$ is the virtual water trade flow from region r to region s in the Yellow River region, $VWT^{s}$ is the virtual water input from other regions from the provinces in this region, and $VWO^{r}$ is the virtual water output of provinces in this region to other regions.

#### 2.2.4. Water Stress Index and Pull Coefficient

This study aims to determine the relationship between virtual water implied by regional trade and local water-resource carrying capacity in the Yellow River region. Based on the research results of Pfister et al. [78], this paper introduces the water stress index, indicating the shortage of water resources in this region, and then analyzes the external dependence of virtual water in each region. Synchronously, this research incorporated the research of Hong et al. [79] and Boero R. et al. [80], using the pull coefficient to explain the influence of the increase of water usage in a single sector on the water usage among all sectors to reveal the virtual water flow law among all sectors in various regions.

The water stress index (*WSI*) is derived from the ratio of total freshwater extraction to total available freshwater in this region annually [78,81]. This index can measure the degree of the lack of water in a region, thus measuring the external dependence of water usage in the region [37,78,81]. The calculation formula is:

(13) $$WSI=\frac{1}{1+e^{-64*WTA((1/0.01)-1)}}$$

The annual freshwater extraction is measured by the ratio of the *WTA* to the annual freshwater supply of different types of users (industry, agriculture, and households) in the region. It can be seen that *WSI* = [0, 1]. Referring to the research results of Pfister et al. [80], when *WSI* is 0, it means there is no water stress, which indicates that water resources are abundant; when *WSI* is 1, that is, the maximum water stress, it means that there is a serious shortage of water resources. A water stress threshold of 0.5 is considered to be moderate.

The pull coefficient $p_{i}^{r}$ refers to the increased degree of water usage by all sectors when the unit water usage of an industrial sector increases, which can be employed to reflect the contribution degree of the water usage of a sector to the increase in the water usage of the whole society. The calculation is as follows:

(14) $$p_{i}^{r}=q_{i}^{r}/y_{i}^{r}$$

where, $p_{i}^{r}$ represents the pull coefficient of i industrial sector in region r, $q_{i}^{r}$, and $y_{i}^{r}$ represents the coefficient of total water usage and direct water usage coefficient of i industrial sector in region r, respectively. It can be seen that, when $p_{i}^{r}$ ≥ 1, the closer the economic ties between sectors are, and the closer the pull coefficient is to 1, and vice versa, the greater the pull coefficient is.

### 2.3. Data Source and Processing

The input–output data of the Yellow River Basin applied in this paper are drawn from the interregional input and output tables (42 sectors) of a total of 31 provinces (autonomous regions and municipalities) domestically in 2015, as compiled by China Carbon Emission Database [82]. The entire water usage dataset, GDP data, and the actual water usage of the agricultural sector of each province (region) are all taken from *China Statistical Yearbook.* The actual usage data of the industrial sector comes from the *2008 China Economic Census Yearbook.* Due to the age of the data, this paper takes the GDP growth rate from 2008 to 2015 as the growth rate of actual water usage, and obtains the water usage reported in the *China Statistical Yearbook* in 2016. Considering the total industrial water usage of each province (region), the total industrial water usage of each province (region) in 2015 was calculated [83,84,85,86].

In terms of the provincial (regional) water-resources bulletin, the actual water usage of the service industry is the urban public water usage in each province. The water usage of each subdivided service-industry sector is based on a proportion of output value along with the domestic water usage of the residents [87,88]. According to the water usage data of the actual industrial sectors of each province (region) in the Yellow River region and the attributes of every industrial sector, and referring to the sector consolidation methods of scholars such as Shi et al. [89] and Chen et al. [90], the 42 sectors in the input–output table have been consolidated into 7 sectors (Table 3).

## 3. Results and Analysis

### 3.1. Virtual Water Usage of Different Sectors in the Yellow River Basin

#### 3.1.1. Analysis of Sectoral Water Usage Coefficient

**Figure 3:**
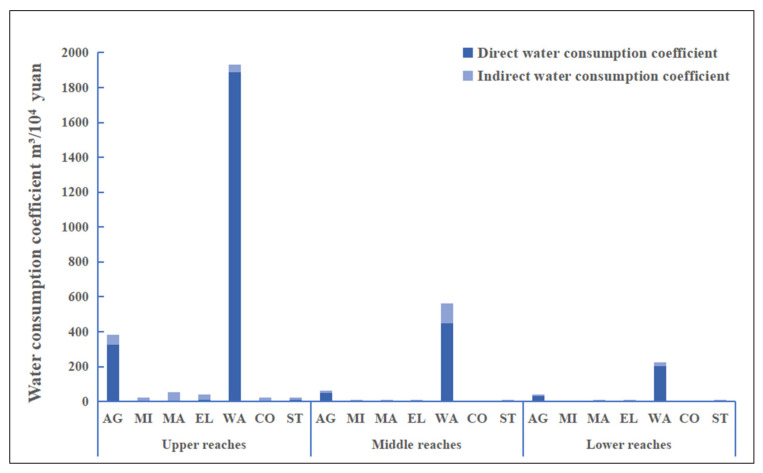
Water consumption coefficient in upper, middle, and lower reaches.

Figure 3 shows the composition of the complete water usage coefficient of 7 industrial sectors in the lower, middle, and upper reaches of the Yellow River. Within the Yellow River Basin, it can be seen that certain differences exist in the total water usage coefficients of a variety of industrial sectors. The total water usage coefficient is the largest in the upstream area and the smallest in the downstream area. The combined upstream, middle, and downstream water usage coefficient of the water supply industry turns out to be the highest, followed by that of agriculture. Specifically, the complete water usage coefficient of the water supply in the upper reach’s industry is more than 1800 m^3^/10,000 RMB, while that of agriculture in the upper and middle reaches is less than 1/5 of the water supply industry. The complete water usage coefficient of other industrial sectors is small, not exceeding 150 m^3^/10,000 RMB. In the upper reaches, the agricultural water usage coefficient is the highest, close to 400 m^3^/10,000 RMB, while those of the middle and lower reaches are less than 100 m^3^/10,000 RMB, indicating that in the industrial structure of the Yellow River Basin, the upper reaches pay more attention to agricultural development than the middle and lower reaches.

#### 3.1.2. Volume of Trade in the Sector of Virtual Water

**Figure 4:**
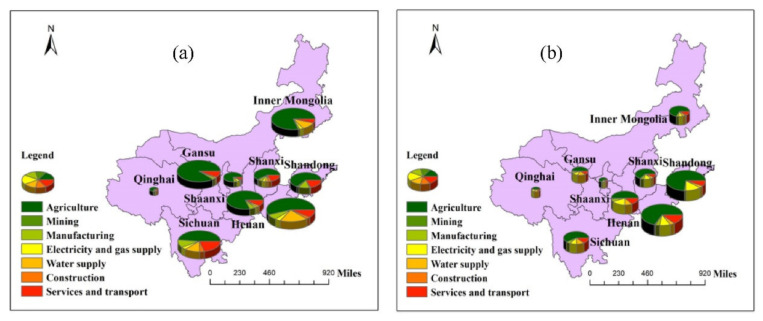
Trade structure of virtual water (%): (**a**) indicates the percentage of industrial sector output; and (**b**) indicates the percentage of industrial sector input.

**Figure 5:**
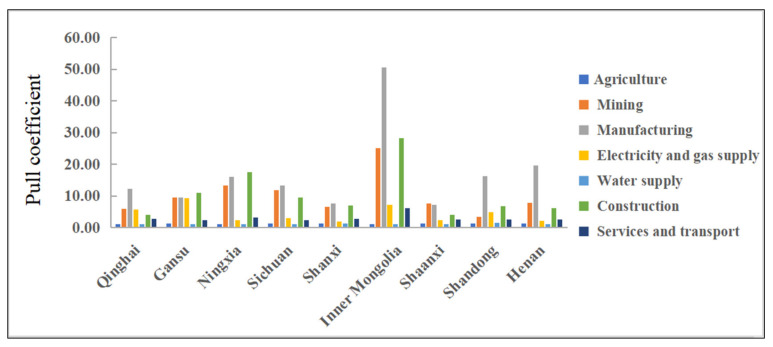
Pull Coefficient of different industrial sectors in different regions.

In all provinces (regions) of the Yellow River region, the virtual water input and output of the industrial sectors was calculated (Figure 4 and Figure 5), and the water flow varied greatly among sectors. First of all, a substantial amount of virtual water is input into and output from agricultural production within the region. It is the largest industrial sector in input and output, accounting for more than 46%, which also determines that the Yellow River Basin is in a state of serious water usage and low regional GDP. The virtual water inputs of the manufacturing, electrical supply, water supply, construction, and service and transportation sectors also account for a large proportion.

Nevertheless, except for manufacturing and service and transportation, there is no corresponding virtual water volume output, indicating that the utilization ratio of virtual water in these sectors is low and the utilization structure of water resources is unreasonable. Secondly, the proportion of the mining industry sector in virtual water input and output is not high, which is related to the concept of ecological protection and transformation and development strongly advocated by the state in recent years.

In terms of regions, in the upstream region, Sichuan Province is the only province whose agricultural virtual water output accounts for less than 70%, only 48.10% of the total, and water resources can be efficiently utilized under favorable circumstances. The virtual water output of the service and transportation and mining industry sectors in Sichuan Province accounts for 19.43%, which is the highest level in the Yellow River Basin, indicating that the tertiary industry in Sichuan Province has frequent foreign exchanges. This phenomenon is also one of the reasons for the high GDP and abundant water resources in Sichuan Province. Gansu Province is the province with the largest proportion of agricultural output, accounting for 88.14%.

However, the high water stress index in Gansu indicates that there is extreme water scarcity in the area. Continuing to export a large number of water-intensive products will make water resources in Gansu even more scarce. In addition to the agricultural sector, the virtual water input sector in Qinghai Province is mainly concentrated on the electric power, water supply, and service and transportation industries. The total proportion of virtual water input in these 3 sectors is 48.65%, which is slightly higher than that in the agricultural sector. Nonetheless, the GDP of Qinghai Province is not high, demonstrating that these sectors have an insufficient utilization of virtual water. In the middle reaches, the province with the largest input of virtual water in the manufacturing industry is Shanxi Province, but its sectoral virtual water output is less than the input, showing that Shanxi Province has not effectively utilized virtual water input to develop the manufacturing industry. Simultaneously, the virtual water output of the service and transportation industries in Shanxi Province account for a large proportion, which can continue to develop. Shaanxi’s agricultural virtual water output also accounts for a large proportion, second only to Gansu Province. However, Shaanxi Province has less rain and more sunshine, poor vegetation distribution, and a poor agricultural development environment.

A large proportion of agricultural virtual water output will aggravate the lack of water resources in Shaanxi Province, as well as destroy the ecology’s overall coordination. In the lower reaches, Shandong Province possess the leading input of agricultural virtual water, which is also the province with the largest input of virtual water in the agricultural sector of the whole Yellow River Basin, indicating that most of the input virtual water in Shandong Province is used for agriculture, which is not conducive to the development of other sectors. However, the output of virtual water in the manufacturing and service and transportation sectors in Shandong Province accounts for 33.17%, making it quite easy to create a higher GDP.

#### 3.1.3. Pull Coefficient Analysis

The pull coefficient is used to further analyze the contribution of the virtual water of various sectors in the Yellow River region to the virtual water of the whole sector, and subsequently evaluate the degree of connection between various sectors of the Yellow River region’s virtual water. The pull coefficient of each industrial sector in every province (region) of the Yellow River Basin is calculated through Formula (14) (Figure 5). As can be seen, the agriculture and water supply sectors have the lowest pull coefficients among all of the provinces (regions) in the Yellow River Basin, with values lower than 1.5, indicating that the economic relationship between these sectors and other sectors is not close. The average pull coefficients of the manufacturing, mining, and construction sectors is obviously greater than that of other sectors, demonstrating that these three sectors have strong economic ties with other sectors, and the increase of unit output in these sectors will require a great deal of virtual water. From the perspective of regional differences, the pull coefficient of the same sector in different provinces (regions) is also quite different. The pull coefficient of the mining, manufacturing, and construction sectors in Inner Mongolia is the largest in the whole Yellow River Basin, which is related to the coal-rich areas of Inner Mongolia. In the future, it will be necessary to speed up the transformation of Inner Mongolia’s resource-based economy, develop a green mining industry, and pay attention to ecological and environmental protection. To alleviate the water shortage in this region, policymakers ought to enhance the water efficiency of the mining industry and strengthen the virtual water input of the manufacturing and construction industries. The pull coefficient of the manufacturing industry in Sichuan, Shandong and Henan Provinces is also large because these provinces mainly rely on the manufacturing industry to drive the rapid growth of the GDP. At the same time, these provinces are virtual water export areas of the manufacturing industry, thus the high value-added manufacturing industry should be developed. The pull coefficient of the manufacturing industry in Ningxia and Qinghai Provinces is also relatively large, but their GDPs lag far behind that of Shandong and Henan Provinces, indicating that a large number of water resources are wasted in the manufacturing sectors in the Ningxia Region and Qinghai Province. Therefore, the differences in the pull coefficients of all the sectors in the same province (region) can be explained by the difference in the number of employees in those sectors in terms of raw material input and production technology level. When a sector operates in two different provinces (regions), its pull coefficient differs due to the differences in water-use efficiency between the local and intermediate rivers.

### 3.2. Regional Virtual Flow Pattern in the Yellow River Basin

#### 3.2.1. Trade Volume of Virtual Water

**Figure 6:**
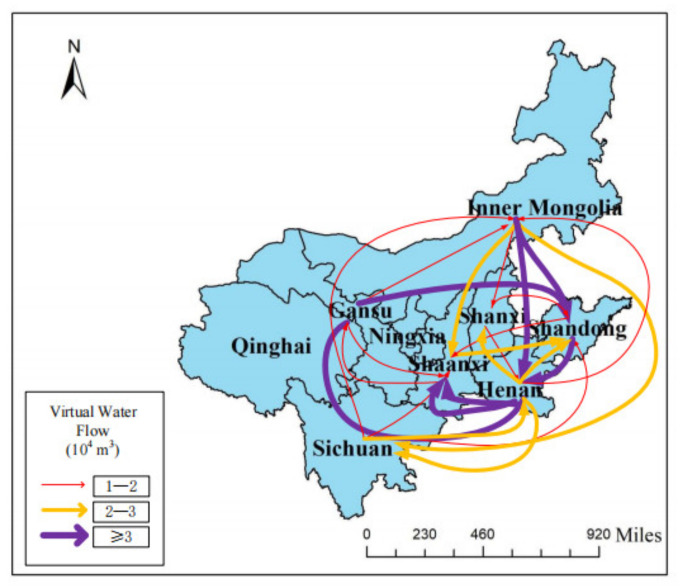
Total virtual water flow of each province in the Yellow River Basin. Note: This figure only shows the flow direction of virtual water trade flow greater than 10^4^ m^3^.

**Figure 7:**
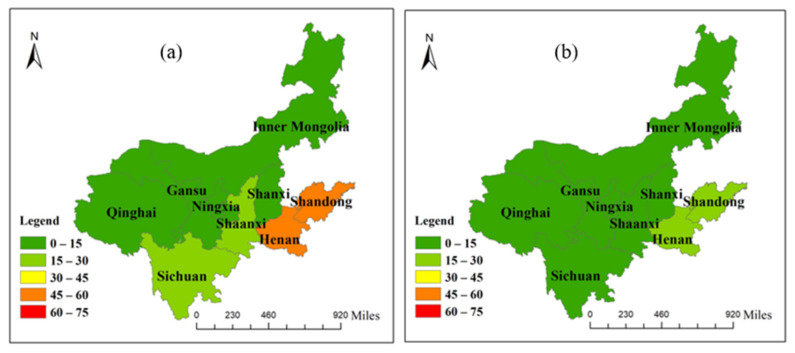
Virtual water inflow of each province in the Yellow River Basin: (**a**) inflow from provinces outside the Yellow River Basin; (**b**) inflow within the Yellow River Basin.

**Figure 8:**
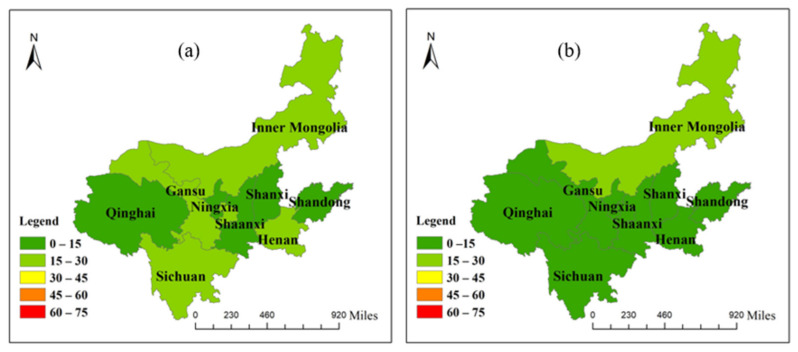
Virtual water outflow of each province in the Yellow River Basin: (**a**) outflow into provinces outside the Yellow River Basin; (**b**) outflow into the Yellow River Basin.

**Figure 9:**
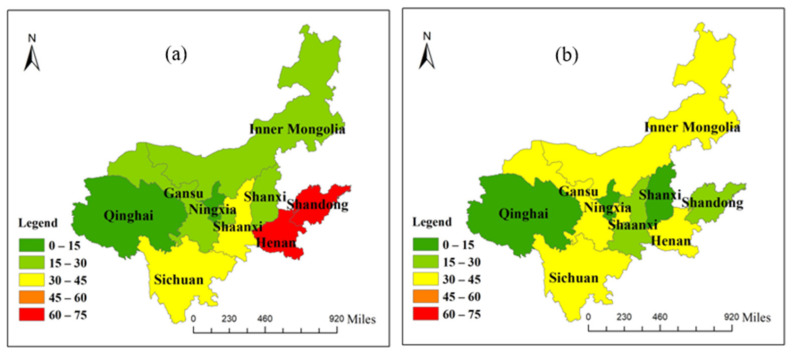
Virtual water trade of each province in the Yellow River Basin: (**a**) the total outflow of virtual water; (**b**) the total inflow of virtual water.

The trade volume of each province (region) in the Yellow River region is calculated according to the input–output multi-regional model of the Yellow River Basin (Figure 6, Figure 7, Figure 8 and Figure 9). It can be seen that the total input and output of virtual water in this region are 27.45 billion m^3^ and 18.64 billion m^3^, respectively, which are in a virtual water net input state, with a total net input of 8.81 billion m^3^. The input of virtual water from the outside of this region is 2.61 times that of the local input, while the external virtual water flow is 1.45 times that of this region, which leads to prominent conflicts between the demand and supply of water resources, and that is not conducive to regional ecological balance. In general, the net input areas importing from other places are mainly concentrated in the midstream and downstream, and the net input virtual water accounts for 96.22% of the total net input of virtual water, reflecting the lack of rationality of water-resource utilization in the region. Among them, Ningxia, Gansu and Inner Mongolia are in the area of virtual water net outflux, and the rest belong to the area of virtual water net influx.

#### 3.2.2. Analysis of Virtual Water and Water-Resource Carrying Capacity

**Figure 10:**
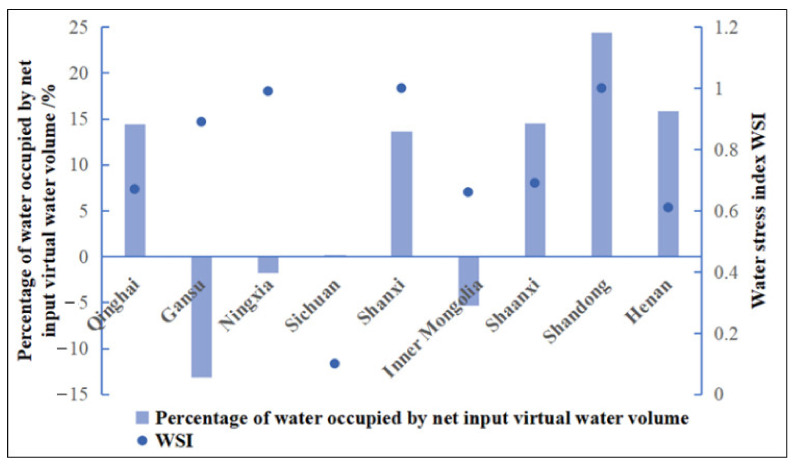
Virtual water external dependence and water stress index.

According to Formula (13), it is possible to calculate the water stress index of each province (region) in this area. Except for Sichuan Province, which has a of *WSI* = 0.1, which belongs to a region rich in water resources, the *WSI* of the other 8 provinces (regions) in this region are between 0.6–1, which corresponds to regions that are seriously short of water (Figure 10). Specifically, the *WSI* of Shandong and Shanxi Provinces are the maximum value of 1, showing that the water shortage is serious, and a large amount of virtual water must be input to alleviate the local water pressures, among which the net input of virtual water in Shandong Province accounts for the largest proportion, and the external dependence of virtual water is about 1/4. The *WSI* of Gansu and Ningxia Provinces are close to 1, indicating that water resources are not abundant, but they output virtual water. The serious disharmony between *WSI* and the virtual water trade will aggravate the shortage of local water and further worsen the ecology. In regions with severe water stress, the lowest *WSI* value is the 0.61 of Henan Province, but the net input of virtual water is second only to Shandong Province, meaning that water resources are not fully utilized, and a large number of water resources are wasted. There is little difference in the *WSI* of Inner Mongolia and Shaanxi Province. However, Shaanxi Province is a virtual water net inflow area, which can alleviate the pressure of insufficient local water resources. In the meanwhile, the Inner Mongolian region should speed up its trade links with other regions to alleviate the water pressure in this region, which would have vital practical significance for the sustainable development of water resources and ecological protection. Sichuan Province is the only region in this region where the *WSI* is lower than 0.5, at only 0.10. Water resources in Sichuan Province are quite abundant, but the net input virtual water accounts for 0.28% of the virtual water. Therefore, the directors of Sichuan Province should appropriately increase the output of virtual water, and increase trade links within and outside the region, which is conducive to improving the water ecological balance in the region.

### 3.3. Virtual Flow Pattern in the Yellow River Basin

**Figure 11:**
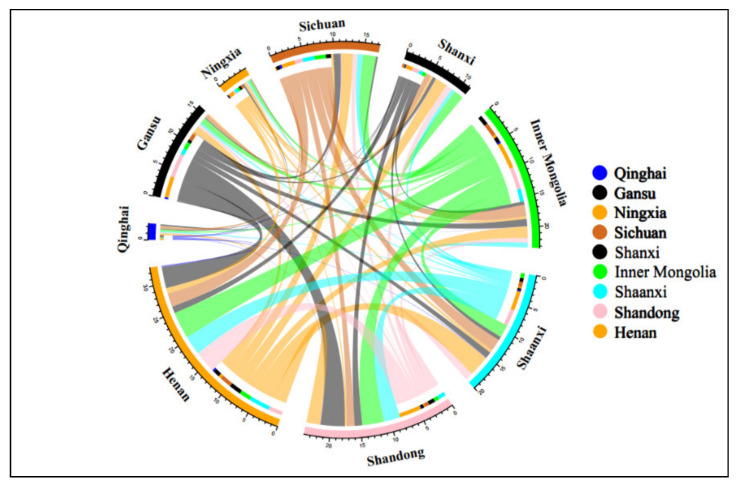
Virtual water flow in the Yellow River Basin (100 million m^3^).

From the virtual water flow table of the Yellow River Basin (Figure 11), it can be seen that the virtual water trade flow in the Yellow River Basin is closely related to geographical location. In the upstream area, with the exception of Qingdao Province, the virtual water output from other provinces in the Yellow River Basin is greater than the virtual water input, which is in the net outflow area of internal virtual water. Among them, Gansu and Inner Mongolia are the provinces with the largest net output of virtual water, and they are the two major sources of virtual water in other provinces (regions) within the Yellow River Basin. However, it should be noted that the *WSI* in Gansu Province is too high, and it is easy to cause a water shortage and damage the ecological environment by excessively providing virtual water to other provinces. The middle and lower reaches are virtual water net input regions, and the virtual water net input of Shandong and Henan Provinces in the lower reaches ranks among the top two in the region, which is consistent with the level of economic development.

Virtual water trade between provinces in the upper reaches is not frequent, and the province with the largest trade flow is Sichuan Province, which is conducive to its good economic development. In the middle reaches, the trade volume of virtual water in Shanxi and Shaanxi Provinces is less than 70 million m^3^, so the trade exchange needs to be strengthened. The interprovincial virtual water-trade flow in the downstream region is large, which is conducive to the common economic development of Shandong and Henan Provinces. In addition to strong trade exchanges between the upper region of Sichuan and Inner Mongolia, as well as the midstream and the upstream, the virtual water-trade flow between other provinces in the upper reaches and the Shaanxi and Shanxi Provinces is small, which is inconducive to economic exchanges and development.

The virtual water trade between the upstream and downstream areas mainly comes from the virtual water output from Gansu and Inner Mongolia to Henan and Shandong Provinces, of which the virtual water output from Gansu Province to the downstream accounts for 63.2% of its total output. The virtual water trade exchanges in the middle and lower reaches are also more frequent, and the virtual water flow basically flows from the middle reaches to the lower reaches. Therefore, it is necessary to strengthen the virtual water trade exchange between the upper, middle, and lower reaches, and make use of the downstream economy to drive the coordinated economic development of Qinghai, Ningxia, Gansu, and Inner Mongolia, so as to achieve the aim of the rational utilization and common development of water resources in all the regions and provinces of the Yellow River Basin.

## 4. Discussion

Virtual water trade can promote economic ties between regions because the importation of virtual water will depend on the water resource endowment of export regions. With increasingly greater implementation of the virtual water strategy, each region becomes increasingly dependent on water resources in other regions [91,92]. Virtual water net import is obviously affected by water resource endowment, and regions with good water resource conditions tend to export virtual water to other regions [30,85]; this is also in line with the theory of comparative advantage in trade. Our results show that the Yellow River Basin is a virtual water net import region because the Yellow River Basin faces serious water shortage, which also confirms that regions with poor water resources usually import virtual water to relieve local water pressures [31]. From within the Yellow River Basin, the export status of virtual water in Gansu Province and Ningxia Autonomous Region is in contradiction with local water resource endowment. However, relevant studies [31,92,93,94] have also shown that northern China, which is seriously short of water, still exports virtual water to southern China through the grain trade, resulting in northern China becoming the main export area of virtual water [95]. Therefore, the method of coordinating the management of water resources in water-shortage areas is still an important research topic for the future. Therefore, this paper puts forward measures for pricing and managing the water resources in water-shortage areas, so as to stimulate the importation of water-intensive commodities and reduce the pressures on local water resources.

The results of this study show that the unreasonable industrial structure in the Yellow River Basin has led to the transfer of local water resources, and the agricultural sector is the largest water-resource import and export sector (Figure 3), which is consistent with the conclusions of relevant studies [96,97]. On the one hand, agriculture is a high water-consumption industry [98], which consumes a lot of blue and green water resources in the process of crop evapotranspiration [99]. Secondly, the Yellow River Basin is an important grain production base in China. In 2020, the total grain output of the Yellow River Basin reached 239 million tons, accounting for 35.6% of the total grain output in China. Farmland irrigation water consumption alone will reach about two-thirds of the water consumption. The backwardness and lack of agricultural irrigation facilities will further increase the consumption and waste of water resources [100]. In addition, the water demands of various departments in a region often compete with one another, forming the characteristics of virtual water trade between regions [72]. For example, the competition between industrial water, service water, ecological environmental water, and agricultural water is more prominent in the Yellow River basin where water resources are scarce. The proportion of agriculture and water-intensive industries in the Yellow River Basin is very high, and the phenomenon of “competing for water” between economic development and ecological protection is very prominent, which is also consistent with the relevant research conclusions [33,35,101]. On the other hand, from the perspective of external factors, climate change may affect agricultural water use in various ways, especially through the changes in temperature and precipitation and the intensification of the frequency and degree of extreme climate events, which will significantly affect the available amount and quality of agricultural water and thus crop-water demand. As a result, agriculture is highly vulnerable to climatic conditions and natural disasters (such as droughts and floods), which inevitably affect interregional food-trade and water-use plans [30,102]. Climate change will not only exacerbate water shortages, but also reduce crop yields, thereby increasing the water footprint [103,104]. Therefore, in terms of agricultural water-use performance management in the Yellow River Basin, combined with high standard farmland construction, promoting large- and medium-sized irrigation areas, building modern irrigation facilities and developing water-saving agricultural technology will reduce water resource consumption. In addition, our recommendations include: optimizing and adjusting the crop planting structures, determining the agricultural industrial structure and planting structure according to local conditions, strictly controlling the planting area of high water-consumption crops, expanding the planting proportion of water-saving and drought-tolerant crops, selecting and promoting new varieties of drought tolerant crops, appropriately implementing rotation fallow, actively developing rainwater harvesting and irrigation, enhancing the capacity of water storage and moisture conservation, improving water-use efficiency, and reducing waste.

In addition, related studies have shown that virtual water flow is closely related to geographic distribution and economic development [105,106,107], which is also confirmed by our study. The virtual water trade volume of Shandong and Henan in the lower reaches of the Yellow River Basin is large, followed by Shanxi and Shaanxi in the middle reaches, and the virtual water trade volume of Qinghai and Ningxia in the lower reaches is the smallest. The advantages of opening to the outside world and strong agricultural development make the virtual water trade volume of Shandong and Henan higher. Qinghai and Ningxia in the downstream region are not only geographically remote, which is not conducive to economic ties, but they also have no obvious industrial advantages. Therefore, the momentum of cross-provincial virtual water flow between products is small. The study also found that the virtual water trade volume of the manufacturing, electrical supply, water supply, construction, and service and transportation industries is high, while the virtual water trade volume of mining is low, which is closely related to the ecological protection and “double carbon” strategic measures proposed by China in recent years. Relevant studies also show that water for power generation is increasing year by year, and the impact on scarce water resources in the basin is becoming more and more serious. In addition, the manufacturing sector [78], construction sector, [108] and service sector [109] are also key industries in the water-saving sector. Therefore, from the perspective of the Yellow River Basin as a whole, strengthening trade links between economies can improve water-use efficiency and optimize the water-use structure of key industries, which will be an important way to alleviate the contradiction between the supply and demand of the water resources of the Yellow River.

Distinguishing and calculating the virtual water flow of green water, blue water, and gray water is helpful for accurately implementing management policy regarding water resources. Because calculating virtual water in this paper involves various industrial sectors in nine regions or provinces of this region, considering the limitation of data acquisition and the inconsistency of differential calculations, only the virtual water flow of blue water has been calculated. The research results are important for formulating water resource policies, but they still need to be further deepened. In addition, because the input–output table is “competitive”, the distinction between domestic and international intermediate inputs is not considered. Constructing a more accurate, interregional input–output table and considering virtual water cross-border transfer in the region is the direction of further research.

## 5. Conclusions

### 5.1. Conclusions

The efficient and rational utilization of water resources is an essential part of protection ecologically. Studying the virtual water usage and flow in water shortage areas is helpful to obtaining an ideal water-resource allocation [110]. By constructing a multi-regional input–output model of the Yellow River Basin, this paper calculates and analyzes the virtual water trade and flow pattern of interregional and intraregional industrial sectors in this region in 2015, and draws the following conclusions:

(1) The whole Yellow River region is in a net input state of virtual water. Among them, the upstream areas—Gansu, Inner Mongolia, and Ningxia Province—are in the net output provinces (regions), while the other six provinces belong to the virtual water net input regions. Gansu’s virtual water input and output state is the most seriously incompatible with the local water-resource carrying capacity among all the provinces discussed.
(2) Agriculture is the largest import and export sector of all regions. In addition to agriculture, the upstream region is sufficient in water resources. The main export sector of virtual water is the water supply industry, and those for the middle- and downstream regions are the services and transportation and manufacturing industries, respectively. Obvious differences exist in the pull coefficients of the same sectors in various provinces (regions). On the whole, the average pull coefficients of mining, manufacturing, and construction are large. The water management of these sectors is conducive to rapid water-resource regulation and rational utilization in this region.
(3) The virtual flow of the Yellow River Basin has obvious geographical distribution characteristics. The trade volume of virtual water in the downstream region is large. The volume of virtual water trade within the upper reaches is low, and the trade links with the middle and lower reaches are insufficient. Henan and Shandong Provinces are the main flow directions in the Yellow River Basin, and Gansu and Inner Mongolia are the dominant virtual water sources.

### 5.2. Suggestions

From the perspective of planning the utilization of water resources nationwide, the research findings suggest reducing water pressures and virtual water-flow imbalances. The nationwide allocation of water resources and the rational use of precipitation in the territory can not only prevent floods and droughts, but also bring a sufficient water supply to China’s industrial and agricultural development and residents’ lives. It also has a certain value in transportation and power generation. There is little difference between the topography of North and South China, and water resources can be mobilized from north to south. The Beijing–Hangzhou canal and the middle route of the South-to-North Water Transfer Project are examples of the North–South distribution of water resources in China. In addition to these two major projects, China can also carry out the large-scale networking of rivers, lakes, and other water areas across the country to make them interconnected. In the case of a flood in a certain place, the excess water could be transferred to another area with fewer water resources. In the case of a drought in a certain place, the water resources of other areas could be mobilized to supplement it, so as to avoid the waste of water resources and maximize the utilization of water resources. According to the decision-making process and the deployment of water-resource management in China, the multi-functional properties of water resources could be fully accomplished in the near future. Under the overall framework of the distribution scheme of the available water supply of the Yellow River, taking into account the ecological water demand, sediment transport volume, external water transfer volume, and water-use structure of provinces along the Yellow River Basin, a joint water-supply pattern of the Yangtze River and the Yellow River will be formed, a dynamic water-distribution scheme of the Yellow River Basin will be constructed, the water-right transfer and compensation system will be gradually improved, and the linkage mechanism between water-use indicators and land indicators will be explored. This will result in comprehensively coordinating the relationship between water, energy, and food; limiting water use for fossil energy development; improving the utilization efficiency of agricultural water resources; building a wind–water complementary power generation system, and implementing the transmission, storage, and utilization of hydrogen energy at normal temperatures and pressures. Ideally, water-scarce regions will import water-intensive products to meet the production and consumption of the region, rather than relying on local production, so as to protect the domestic water resources. In this case, the water resources required by the whole production chain actually come from export to import regions through interregional trade.

According to the research results mentioned above, this paper puts forward the following suggestions in order to achieve the rational allocation of water resources:

(1) China should vigorously implement the ecological compensation policy of water usage. Although the region is in the virtual water net input area as a whole, the *WSI* of Gansu and Ningxia is high, which is seriously inconsistent with the virtual water net output state. The utilization of water resources should be distributed comprehensively throughout the country. By reducing the virtual water flow in Gansu and Ningxia, the local ecological development and water resource allocation balance can be protected. China should also appropriately increase the output of virtual water in Sichuan and grasp the advantages of local green water resources. Meanwhile, we recommend increasing the virtual water output from other surplus provinces to Henan and Shandong Provinces, reducing the pressures of water outflow, and ensuring local water safety and ecological security.
(2) All industry sectors should adhere to the principle of “determining production by water”. The whole Yellow River Basin should develop water-saving agricultural techniques, change the traditional mode of agricultural production, strictly control the total water, and improve water-usage efficiency. The upper reaches of the Yellow River Basin should enforce the technological innovation investment and water-use efficiency, and the regional water-shortage situation should be alleviated by importing water-intensive products to water-rich areas; The middle reaches should speed up the transformation to a resource-based economy, develop water-saving industries, and vigorously develop the service and transportation industry; The lower reaches should speed up the development of high value-added manufacturing industries and strengthen economic ties inside and outside the region.
(3) China should fully strengthen the exchanges and cooperation between the lower, middle, and upper reaches, and actively explore the institutional mechanism of water ecological protection. China should establish an internal, cooperative development mechanism in this region with the goals of common economic development, water conservation, and ecological protection. Through trade-oriented interprovincial cooperation, China should reduce the intermediate links of ineffective water use, make the virtual water flow to the most needy regions and sectors, improve water sewage efficiency, and drive economic development. China should also comprehensively improve the interprovincial virtual water-trade flow, give full play to the economic ties between the lower, middle, and upper reaches, and jointly realize the sustainable development of the economy, as well as the ecological environment.

## Figures and Tables

**Table 1:** Water Resources and economic development of the Yellow River Basin in 2019 (source: *China Statistical Yearbook* (2020) and *China Water Resources Bulletin* (2020).

Province (Region)		Total Water Resources/100 Million m^3^	Total Water Consumption/100 Million m^3^	GDP/100 Million Yuan
Upper reaches	Qinghai	919.30	26.20	2965.95
	Gansu	325.90	110.00	8718.30
	Ningxia	12.60	69.90	3748.48
	Sichuan	2748.90	252.40	46,615.82
	Inner Mongolia	447.90	190.90	17,212.53
Middle reaches	Shanxi	97.30	76.00	17,026.68
	Shaanxi	495.30	92.60	25,793.17
Lower reaches	Shandong	195.20	225.30	71,067.53
	Henan	168.60	237.80	54,259.20
The Yellow River Basin		5411.00	1281.10	247,407.66
Whole country		29,041.00	6021.20	986,515.20
Percentage of Yellow River Basin in China		18.63%	21.28%	25.08%
Percentage of upper reaches in the Yellow River Basin		82.32%	50.69%	32.04%
Percentage of middle reaches in the Yellow River Basin		10.95%	13.16%	17.31%
Percentage of lower reaches in the Yellow River Basin		6.72%	36.15%	50.66%

**Table 2:** Summary table of multi-regional input–output in the Yellow River Basin.

Item			Intermediate Use				Final Demand		Export	Total Output
			Qinghai	…	Henan	Other Regions	Qinghai … Henan	Other Regions		
			Sector1 … Sector42	…	Sector1 … Sector42	Sector1 … Sector42				
Intermediate input	Qinghai	Sector1	$z_{1,1}^{1,1}$ … $z_{1,42}^{1,1}$	…	$z_{1,1}^{1,9}$ … $z_{1,42}^{1,9}$	$z_{1,1}^{1,10}$ … $z_{1,42}^{1,10}$	$f_{1,1}^{1}$ … $f_{1,1}^{9}$	$f_{1}^{1,10}$	$e_{1}^{1}$	$X_{1}^{1}$
		…	…	…	…	…	…	…	…	…
		Sector42	$z_{42,1}^{1,1}$ … $z_{42,42}^{1,1}$	…	$z_{42,1}^{1,9}$ … $z_{42,42}^{1,9}$	$z_{42,1}^{1,10}$ … $z_{42,42}^{1,10}$	$f_{42}^{1,1}$ … $f_{42}^{1,9}$	$f_{42}^{1,10}$	$e_{42}^{1}$	$X_{42}^{1}$
	…	…	…	…	…	…	…	…	…	…
	Henan	Sector1	$z_{1,1}^{9,1}$ … $z_{1,42}^{9,1}$	…	$z_{1,1}^{9,9}$ … $z_{1,42}^{9,9}$	$z_{1,1}^{9,10}$ … $z_{1,42}^{9,10}$	$f_{1}^{9,1}$ … $f_{1}^{9,9}$	$f_{1}^{9,10}$	$e_{1}^{9}$	$X_{1}^{9}$
		…	…	…	…	…	…	…	…	…
		Sector42	$z_{42,1}^{9,1}$ … $z_{42,42}^{9,1}$	…	$z_{42,1}^{9,9}$ … $z_{42,42}^{9,9}$	$z_{42,1}^{1,10}$ … $z_{42,42}^{9,10}$	$f_{42}^{9,1}$ … $f_{42}^{9,9}$	$f_{42}^{9,10}$	$e_{42}^{9}$	$X_{42}^{9}$

**Table 3:** Input–output table: Detailed list of 42 combined sectors.

Combined 7 Sectors	Department Abbreviation
Agriculture	AG
Mining	MI
Water supply	WA
Electricity and gas supply	EL
Manufacturing	MA
Construction	CO
Services and transport	ST

## Data Availability

The input-output table data are from *China’s carbon accounting database*, and the water use and GDP data can be obtained from *China’s statistical yearbook*.
